# The Impact of Glucose on Corticospinal and Intracortical Excitability

**DOI:** 10.3390/brainsci9120339

**Published:** 2019-11-25

**Authors:** Stephen L. Toepp, Claudia V. Turco, Mitchell B. Locke, Chiara Nicolini, Roshni Ravi, Aimee J. Nelson

**Affiliations:** Department of Kinesiology, McMaster University, Hamilton, ON L8S 4K1, Canada; toeppsl@mcmaster.ca (S.L.T.); turcocv@mcmaster.ca (C.V.T.); lockemb@mcmaster.ca (M.B.L.); nicolinichiaratn@gmail.com (C.N.); roshniravi67@gmail.com (R.R.)

**Keywords:** glucose, SICI, SAI, LAI

## Abstract

Neurotransmission is highly dependent on the availability of glucose-derived energy, although it is unclear how glucose availability modulates corticospinal and intracortical excitability as assessed via transcranial magnetic stimulation (TMS). In this double-blinded placebo-controlled study, we tested the effect of acute glucose intake on motor-evoked potential (MEP) recruitment curves, short-interval intracortical inhibition (SICI), short-latency afferent inhibition (SAI) and long-latency afferent inhibition (LAI). Eighteen healthy males participated in four sessions. Session 1 involved acquisition of an individualized blood glucose response curve. This allowed measurements to be time-locked to an individualized glucose peak after consuming one of three drinks during the subsequent three sessions. Participants were administered a 300 mL concealed solution containing 75 g of glucose, sucralose, or water in separate sessions. Dependent measures were assessed at baseline and twice after drinking the solution. Secondary measures included blood glucose and mean arterial pressure. Corticospinal excitability and blood pressure increased following the drink across all treatments. No changes were observed in SICI, SAI or LAI. There was no rise in corticospinal excitability that was specific to the glucose drink, suggesting that acute changes in glucose levels do not necessarily alter TMS measures of corticospinal or intracortical excitability.

## 1. Introduction

Glucose is the brain’s primary energy substrate and provides the main carbon source for de novo synthesis of large compounds required for essential ranging processes, from neurotransmission to the management of oxidative stress [1,2,3,4]. Although the substantial influence of glucose is evident in several clinical contexts [5,6,7], a comprehensive neurophysiological profile has yet to be compiled in healthy humans during periods of fasting versus high-circulating glucose following feeding.

A small number of studies have used non-invasive transcranial magnetic stimulation (TMS) to compare neurophysiological measures in hyperglycemic, normoglycemic or fasting conditions [8,9,10]. Specterman et al. [10] reported a 3-fold increase in the size of motor-evoked potentials (MEPs) 60 min after ingestion of 68 g of glucose, such that greater increases in MEPs were correlated with greater increases in blood glucose levels. Badawy and colleagues [9] observed greater long interval intracortical inhibition (LICI) in epileptic and healthy individuals when in a fed (i.e., two hours after a meal) compared to fasted state (i.e., 12 h overnight). However, not all studies have detected an effect of glucose on TMS measures. Andersen and colleagues [8] manipulated glucose levels in type 1 diabetics via an intravenous glucose pump and did not observe any change in cortical motor thresholds, a common measure of corticomotor excitability. TMS can also be used to probe the sensorimotor system with measures of short- and long-latency afferent (SAI, LAI). To date, no studies have investigated the influence of glucose on afferent inhibition. SAI and LAI are impaired in populations with Alzheimer’s and Parkinson’s disease [11], both of which display altered central glucose metabolism [12,13].

Most research to date that directly tests the effects of glucose on the motor system provides information regarding a limited variety of neurophysiological measurements. For example, the study conducted by Specterman and colleagues [10] measured MEPs using a time-efficient protocol involving the delivery of only 15 suprathreshold TMS pulses to obtain a measure of average MEP size. This approach allows for relatively high temporal resolution but contrasts with more comprehensive tests which could probe underlying mechanisms. For example, acquisition of MEP recruitment curves, while more time consuming, provides information regarding cortical glutamate levels [14]. Investigating a proxy measure of glutamate levels is useful, since glutamatergic neurotransmission has been linked with glucose in in vitro [15,16] and in situ [17] neurobiological studies. It may also be worthwhile to probe gamma-aminobutyric acid (GABA)-mediated intracortical inhibition using paired-pulse TMS. Although Badawy and colleagues found no significant effect of glucose on short interval intracortical inhibition (SICI), all significant and non-significant differences between fasted and fed participants were in the direction of increased inhibition or decreased facilitation after feeding as opposed to after fasting [9]. This is counterintuitive, given the apparent increase in MEP size after glucose ingestion [10], and warrants further investigation.

The goal of this study was to test the effect of glucose ingestion on the healthy human brain using a variety of non-invasive neurophysiological measures which have relevant mechanistic underpinnings. This investigation also controlled for the influence of a sweet placebo and time-locked measurements to individually measured peak blood glucose latencies. It was hypothesized that glucose would increase corticospinal excitability and SICI. The primary observation was that glucose did not change SICI, SAI, LAI or corticospinal excitability. Furthermore, corticospinal excitability and blood pressure increased over time when data were averaged across all treatments.

## 2. Materials and Methods

### 2.1. Participants 

Healthy, young (*n* = 18, 22.8 ± 2.4 years), right-handed, male non-smokers were recruited from the McMaster University student population. Participants passed a screening for TMS contraindications [18] and were identified as right-handed using a modified handedness questionnaire [19]. Inactive individuals were excluded using the International Physical Activity Questionnaire (IPAQ, <600 MET-minutes/week) to reduce the risk of influence from prediabetic impairment of glucose metabolism, which is inversely correlated with physical activity level [20]. This study was approved by the Hamilton Integrated Research Ethics Board (HiREB) and conformed to the declaration of Helsinki.

### 2.2. Electromyography

Motor-evoked potentials were recorded via surface electromyography (EMG) over the first dorsal interosseous (FDI) muscle. FDI was chosen because MEPs from this muscle have demonstrated good inter- and intra-session reliability [21]. Adhesive electrodes (9 mm diameter Ag-AgCl) were placed over the FDI muscle belly and the metacarpal head of the index finger. The EMG signal was amplified 1000x and sampled at 5 kHz with low and high pass signal filters of 2.5 kHz and 20 Hz, respectively. EMG data was recorded using an analog-to-digital interface (Power 1401; Cambridge Electronics Design, Cambridge, UK) in combination with Signal/CED analysis software (Signal version 6.02; Cambridge Electronics Design).

### 2.3. Transcranial Magnetic Stimulation

Participants sat upright in the testing chair with their palms resting supine and elbows at an approximate 45° angle. Single and paired-pulse TMS was delivered with a custom 50 mm figure-of-eight coil, connected to a Magstim Bistim stimulator (Magstim, Whitland, UK). The coil was held over the left primary motor cortex (M1) and the optimal stimulation location or “motor hotspot” was targeted using Brainsight neuro-navigation software (Rogue Research, Canada). The motor hotspot for FDI muscle was determined by delivering pulses at 50% of the maximum stimulator output (%MSO) over the approximate location of M1 while adjusting coil placement until the TMS pulses reliably evoked large MEPs in the FDI muscle. The angle of the coil relative to the midsagittal plane was maintained at 45° to induce posterior-to-anterior current in cortical tissue.

### 2.4. Resting Motor Threshold

Resting motor threshold (RMT) was defined as the stimulus intensity (%MSO) that evokes an MEP (i.e., peak-to-peak amplitude >50 µV) 50% of the time. This value was determined using TMS_MTAT_2.0 freeware (http://clinicalresearcher.org/software.htm). The starting stimulus intensity was set to 37% MSO. Twenty TMS pulses were then delivered over M1, adjusting the intensity after each pulse, as determined by the MTAT software based on the MEP occurrence (or lack thereof) in the previous trial [22].

### 2.5. MEP Recruitment Curve

Corticospinal excitability was measured using single-pulse TMS to obtain MEP recruitment curves. Eight TMS pulses were delivered at 90%, 100%, 110%, 120%, 130%, 140%, 150%, 160%, 170%, 180%, 190%, and 200% of RMT in a randomized order, with an inter-stimulus interval of 4 s. The MEP amplitude was plotted against stimulus intensity and data were fit with a Boltzmann sigmoidal curve. The regression line was segmented 1000 times and the area under the recruitment curve (AURC) was quantified by trapezoidal integration.

### 2.6. Short Interval Intracortical Inhibition

Short-interval intracortical inhibition (SICI) was measured using a paired-pulse TMS protocol. The suprathreshold test stimulus (TS) was adjusted to the intensity that evoked an MEP with a size of approximately 1 mV in peak–peak amplitude. The conditioning stimulus (CS) preceded the TS by 2 ms, with a stimulus intensity of 80% of RMT. Twelve unconditioned (MEPTS) and 12 conditioned (MEPCS-TS) pulses were delivered in a randomized order, with a 5 s inter-trial interval. The magnitude of SICI was quantified using the ratio of conditioned to unconditioned MEP size (MEPCS-TS/ MEPTS).

### 2.7. Afferent Inhibition

Electroencephalography (EEG) electrodes were positioned over C3′ (located 2 cm posterior to C3) and referenced to Fz (International 10–20 system). A bar electrode was positioned over the median nerve at the wrist (cathode proximal) to deliver square wave electrical pulses (0.2 s pulse width) using a constant current stimulator (DS7AH; Digitimer, Welwyn Garden City, UK). Nerve stimulation was delivered at the minimum intensity that evoked a visible twitch in the abductor pollicis brevis (APB) muscle. Time-locked averaging of five hundred stimuli delivered at 3 Hz was used to determine the latency of the N20 peak of the somatosensory-evoked potential (SEP).

To acquire afferent inhibition, the TMS intensity was set to evoke a MEP of ~1 mV in the right FDI muscle. Electrical stimulation was delivered to the median nerve at the wrist at the minimum intensity that evoked a visible twitch in the APB muscle. The average intensity of nerve stimulation was 11.1 ± 3.8 mA. For SAI, the interstimulus interval (ISI) between peripheral nerve stimulation and TMS was 4 ms longer than the N20 latency (i.e., N20 + 4 ms). An ISI of 200 ms was used to acquire LAI. Twelve unconditioned stimuli (MEPTS) were randomly presented among 36 conditioned stimuli (nerve stimulation followed by TMS, twelve stimuli per ISI), with a 5 s inter-trial interval. The magnitude of SAI/LAI was expressed as the ratio of the conditioned to the unconditioned MEP amplitude.

### 2.8. Blood Glucose and Blood Pressure 

Capillary blood glucose measurements were performed via the glucose oxidase method using a hand-held diabetes monitoring device (Abbott MediSense FreeStyle Precision Neo Blood Glucose and Ketone Monitoring System, Abbott). Since previous research has indicated that blood pressure may be elevated by ingestion of a large glucose bolus [23,24], mean arterial blood pressure was measured using an automated blood pressure monitor (OMRON Blood Pressure Monitor, OMRON Healthcare). The mean arterial pressure (MAP) was calculated from the systolic (SBP) and diastolic blood pressure (DBP) as indicated below:

MAP = (2DBP + SBP)/3(1)

### 2.9. Experimental Design

**Figure 1:**
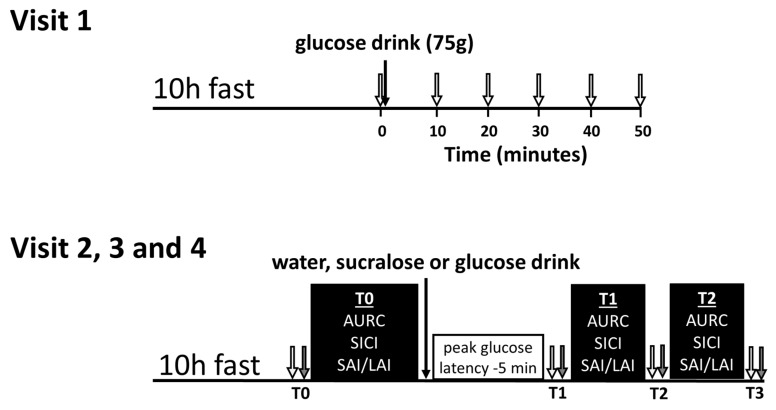
Timeline for the preliminary visit (visit 1) and the three experimental visits (visits 2, 3 and 4). For visit 1, baseline blood glucose was measured (0 min) and then participants drank 75 g of glucose. Next, blood glucose was measured every 10 min for 50 min or until peak was observed (open arrows). For visits 2, 3 and 4, the transcranial magnetic stimulation (TMS) testing bouts (black boxes) at T0 and T1 were separated by a rest period equal to the glucose latency minus 5 min. Mean arterial pressure (grey arrows) and blood glucose (open arrows) were measured before, after and in between the TMS testing bouts and are labeled T0, T1, T2 and T3. TMS—transcranial magnetic stimulation; AURC—area under the recruitment curve; SICI—short-interval intracortical inhibition; SAI/LAI—short-latency afferent inhibition/long-latency afferent inhibition.

This study implemented a double-blinded, three-way crossover design in which fasted participants were assessed before and after ingestion of water, a sucralose-flavored placebo or a 75 g glucose bolus. All solutions were 300 mL. Prior to the first experimental testing session, participants completed a preliminary testing session. A schematic of the study schedule for each participant is shown in Figure 1.

Visit 1 was used to assess a time-course for glucose metabolism, allowing the subsequent TMS measures on visits 2, 3, and 4 to be individualized. Participants arrived in the lab having fasted for a minimum of 10 h, and then ingested a 75 g glucose bolus in 300 mL of solution. Finger-prick blood samples were collected and analyzed at 10 min intervals, as indicated in Figure 1 (top). The latency at which peak blood glucose occurred was used to ensure that TMS tests are conducted during a period of high-circulating glucose for each individual. 

Visits 2, 3 and 4 were scheduled at least 48 h apart. On the day of each visit, participants arrived in the lab having fasted for 10 h and then ingested a 300 mL solution containing either plain water, sucralose-sweetened placebo (5 g/300 mL Splenda^®^ solution), or a 75 g oral glucose tolerance test bolus. TMS measures were acquired before ingestion (T0), 5 min before each participant’s peak blood glucose latency (~30 min after drink ingestion) as determined in Visit 1 (T1), and ~1 h after ingestion, corresponding to the approximated peak of glucose levels in the cerebrospinal fluid (T2) occurring ~30 min after plasma glucose [25]. SAI, LAI, SICI and MEP recruitment curves were measured in a pseudorandomized order which was determined using an online Latin square generator (https://hamsterandwheel.com/grids/index2d.php). Capillary blood glucose and blood pressure were measured before and after each of the post-drink bouts, as denoted by the labels T1, T2 and T3 (see Figure 1, bottom). 

The McMaster University Medical Centre (MUMC) research pharmacy provided a randomized treatment schedule. All treatment solutions were provided in uniform, shrouded bottles, with a letter code corresponding to the order of delivery. MUMC pharmacy held the drink randomization (i.e., drink identity) key until collection was complete to ensure that the experimenters were blind to the identity of the drink. Blood glucose and subjective ratings of sweetness were recorded by an unblinded researcher who did not otherwise take part in data collection or analysis. The sucralose-sweetened placebo was taste-matched with the 75 g glucose solution by MUMC pharmacy. Participants were explicitly asked not to comment on the taste of the drink to the researchers and it was made clear that this was very important to the integrity of the study. The participants were blind to the identity of the drink to the degree that they could not distinguish between the sucralose placebo and the glucose solutions (water was not masked with any taste).

### 2.10. Statistical Analyses

Trials were discarded if the EMG activity was >100 µV in the 100 ms preceding the stimulation artefacts, similar to previous work [26]. Normality was assessed with the Shapiro–Wilks test and a square-root or log transformation was applied in cases where data was not normally distributed. 

First, to confirm that inhibition was observed for measures of SICI, LAI and SAI, two-way ANOVAs with the factors PATTERN (two levels: unconditioned MEP and conditioned MEP) and TIME (three levels: T0, T1, T2) were performed. Next, one-way ANOVAs using the within-subjects factor of TREATMENT (three levels: glucose, sucralose, water) were used to confirm that T0 data were not different between visits. Next, outlier analysis was performed using SPSS. Four outliers were removed from the SICI data and one was removed from the SAI data. Data were subsequently analyzed using repeated-measure ANOVAs with factors TREATMENT (three levels: glucose, sucralose, water) and TIME (three levels: T0, T1, T2). Post-hoc testing was performed with Bonferroni-corrected two-tailed paired t-tests. A Conover’s ANOVA [27] was performed in lieu of a parametric ANOVA in cases where the data was not normally distributed (even after attempted transformations), with the Wilcoxon-signed rank test used for post-hoc testing. Supplementary measures of capillary blood glucose and MAP were assessed using two-way repeated measures ANOVA with four levels of TIME (T0, T1, T2, T3). 

Wilcoxon signed-rank tests were used to assess the hypotheses that glucose would strengthen SICI and increase AURC, as well as all indicated post-hoc comparisons. Effect sizes were calculated using Cohen’s d for paired t-tests and r for Wilcoxon’s tests. Significance for all statistical tests was set to alpha <0.05. Data from TMS, blood glucose and blood pressure measurements are included in the supplementary materials.

## 3. Results

### 3.1. Blood Glucose

The peak plasma glucose concentration after ingestion of the glucose bolus on Visit 1 was 9.5 ± 1.0 mmol/L. This represents a 2-fold increase from the fasting glucose level of 4.8 ± 0.4 mmol/L, with a rise of 4.7 ± 0.9 mmol/L. The majority of participants had peak glucose latencies of 40 min (*n* = 10), followed by six peaking at 30 min, and one participant peaking at 20 min and 50 min each. The observed peak glucose latency spread of 30 min emphasizes the importance of the individualized approach tested herein.

**Figure 2:**
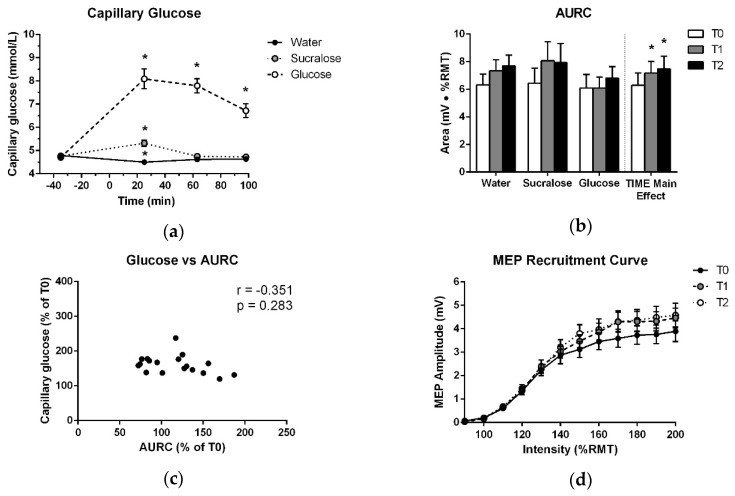
(**a**) Mean and standard error of capillary blood glucose levels over the course of each testing session. Measurements are plotted at each timepoint before and after ingestion of glucose, sucralose or water, which were consumed at 0 min. (**b**) Means and standard errors of AURC data entered into the ANOVA and treatment-averaged data, showing the significant effect of TIME. (**c**) Average relative increase in plasma glucose across T1, T2 and T3, plotted against the average relative increase in AURC across T1 and T2. (**d**) Treatment-averaged motor-evoked potentials (MEP) responses with mean and standard error at each TMS intensity of the recruitment curve for T0, T1 and T2. * indicates a significant difference from T0 (*p* < 0.05).

As expected, glucose levels for the experimental visits exhibited a significant effect of TREATMENT (F (2,17) = 108.261; *p* < 0.001), TIME (F (3,17) = 31.037; *p* < 0.001) and TREATMENT × TIME (F (6,17) = 29.722; *p* < 0.001). In the glucose delivery condition, plasma levels measured at T1 were 3.4 ± 1.9 mmol/L higher than fasting and remained elevated by 2.1 ± 1.2 mmol/L at T3. These data confirm that glucose levels were substantially increased during both post-drink TMS testing bouts in the glucose delivery condition. Notably, there was a slight increase in glucose level (<1 mmol/L) at T1 after the sucralose-sweetened placebo and a slight reduction at T1 following water. However, blood glucose returned to fasting levels at T2 and T3 in both cases. These data are displayed in Figure 2a and *p*-values and effect sizes for significant changes are reported in Table 1.

### 3.2. MEP Recruitment Curve

As shown in Figure 2b, there was no increase in AURC after glucose at T1 or T2 (*p* = 0.110). There was only a significant effect of TIME, which was independent of treatment (Table 2, Figure 2b,d). No other effects in the ANOVA and no association between capillary blood glucose levels and the change in AURC at T1 or T2 were found (Table 3, Figure 2c). Further, no difference was detected between T0 data for the three treatments (Table 4).

### 3.3. SICI

**Figure 3:**
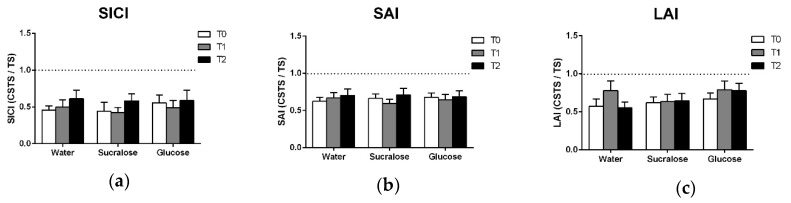
Means and standard errors of (**a**) SICI, (**b**) SAI and (**c**) LAI data. All data are expressed as the ratio of the conditioned response (CSTS) to the unconditioned response (TS) such that the degree to which the ratio falls below 1.0 (i.e., the dotted line) reflects the magnitude of inhibition observed.

The presence of SICI was confirmed by two-way ANOVAs which showed the main effects of PATTERN for each treatment (Table 4), indicating that inhibition was observed at all timepoints. There was no increase in SICI following glucose (Figure 3a) and there was no effect of TREATMENT, TIME or TREATMENT × TIME observed in the ANOVA (Table 2).

### 3.4. Afferent Inhibition

For SAI and LAI, two-way ANOVAs confirmed the presence of significant inhibition (Table 4). There was no effect of TREATMENT, TIME or TREATMENT × TIME (Table 2, Figure 2c and Figure 3b).

### 3.5. Mean Arterial Pressure

**Figure 4:**
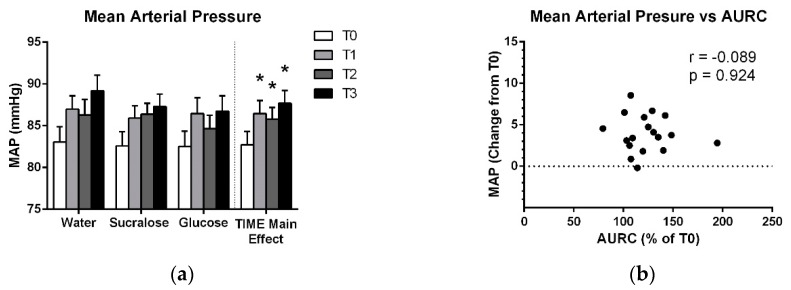
(**a**) Means and standard errors of mean arterial pressure for each treatment and for the treatment-averaged data showing the effect of TIME. (**b**) Average increase in mean arterial pressure across T1, T2 and T3 plotted against the average relative increase in AURC across the T1 and T2 TMS testing bouts. * indicates a significant difference from baseline (*p* < 0.05).

Figure 4 shows a main effect of TIME (F_(2,12_ = 15.119; *p* < 0.001), with significant increases in mean arterial pressure at all post-drink pressure which were observed across all treatments. Post-hoc analysis of treatment-averaged MAP data indicates that this increase from baseline (T0) was evident at T1, T2 and T3 (*p* < 0.001) (Figure 4a). There were also no correlations between the increase in mean arterial pressure across treatments and changes in AURC at T1 or T2 (Table 3).

## 4. Discussion

The primary finding of this study is that glucose did not lead to an increase in corticospinal excitability. Further, there was no change in SICI, SAI or LAI. However, we did observe that all treatments contributed to an increase in corticospinal excitability and mean arterial pressure. This increase in excitability was not related to the increase in mean arterial blood pressure. We will discuss below the lack of glucose effects on TMS measures and the possible role of hydration and prolonged brain stimulation on changes in mean arterial pressure. 

The increase in AURC across all conditions contrasts with previous research reporting an increase in corticospinal excitability following glucose ingestion and not after a no-calorie placebo [10]. Notably, Specterman and colleagues [10] tested a small sample size (*n* = 4) compared to the present study. They also measured corticospinal excitability by quantifying the average size of MEPs evoked at 110% of RMT, while the present study did so across the multiple intensities of a recruitment curve. Therefore, it is possible that the contrasting results reflect a combination of differences in the study population and measurement protocol.

The finding that glucose did not alter SICI is in line with the results reported by Badawy and colleagues [9], who found that SICI was not significantly different after 12 h of fasting versus 2 h after a meal. It should be noted that the authors did report significantly greater LICI after feeding than after fasting. These findings suggest that glucose has a different effect on LICI versus SICI. Indeed, each measure is thought to reflect the activity of different neurotransmitter receptors, with GABA_A_ receptors mediating SICI and GABA_B_ receptors involved in LICI [28,29].

Intake of glucose, sucralose or water did not change measures of SAI or LAI. SAI and LAI are impaired in multiple neurodegenerative conditions, including Alzheimer’s and Parkinson’s disease [30], and have potential clinical utility as diagnostic tools or biomarkers of sensorimotor function. Therefore, it is important to establish whether factors of daily living influence the acquisition of these measures. The present study suggests that neither elevated glucose levels or hydration change the magnitude of SAI or LAI. The results also suggest that the weakening of SAI/LAI in these neurodegenerative populations [12,13] does not reflect the impairment in glucose metabolism. 

Increased AURC at T1 and T2 coincided with a treatment-nonspecific rise in mean arterial pressure, suggesting influence from factors intrinsic to the testing protocol. These factors may include hydration, discomfort with the testing setup (i.e., a white-coat effect) or the delivery of a high number of TMS pulses over M1. Since participants were not asked to dehydrate themselves overnight, and the 300 mL drink is a relatively low volume of fluid, it seems unlikely that hydration was a major factor with respect to mean arterial pressure. It is more likely that changes in emotional state due to fatigue or level of interest, or the repetitive stimulation itself could have contributed to the observed TIME effects. The sensorimotor cortex has functional connections with brainstem structures involved in the regulation of vasomotor tone, which are modulated by cortical stimulation [31]. Transcranial direct-current stimulation of the sensorimotor area has been shown to acutely suppress blood pressure and adrenocorticotropic hormone levels [32]. If TMS changes blood pressure similarly to direct-current stimulation, it is possible that an association between changes in mean arterial pressure and AURC was masked by acute effects of TMS delivered at T1 and T2. In addition, Binkofski et al. [32] described longer-latency changes (1–2 h) in cerebral energy metabolism and glucose uptake following brain stimulation, suggesting that baseline TMS could have masked relationships between glucose and AURC at T1 and T2. This may provide an explanation for the contrasting results with Specterman et al. [10], who delivered only 45 TMS pulses at baseline and 15 per timepoint. They observed a positive association between glucose level and MEP size, while we delivered 180 pulses at T0, T1 and T2 and did not observe the same relationship.

In the present study, the timing of T1 was based on the latency of peak glucose levels obtained on Visit 1. The purpose of this protocol was to maximize the opportunity to observe a change in corticospinal excitability following glucose ingestion. However, the day-to-day variability in glucose metabolism was not assessed. Therefore, while we observed a significant rise in glucose levels following ingestion of the solution in the glucose session, the latency of peak glucose may have varied from day-to-day.

### Future Considerations

It is important for future research to attempt to replicate previously observed effects of glucose on TMS measurements, using testing protocols which consider potential confounding factors such as the number of TMS pulses, session duration and hydration. The effect of TMS pulse load on changes in glucose levels and sympathetic tone should also be investigated to facilitate the interpretation and design of future TMS research. While glucose did not change our TMS measures, other dietary factors such as caffeine consumption [10,33], prolonged fasting [34] and ketogenic diets [35] merit further investigation as these may be important and easily modifiable factors in TMS research.

## 5. Conclusions

The present study found no explicit effect of glucose on corticospinal excitability, intracortical inhibition or afferent inhibition, but corticospinal excitability and mean arterial pressure increased across all treatments over the course of the experiment. These non-treatment-specific increases suggest that TMS measurements could be sensitive to various confounding factors related to repeated magnetic stimulation of the cortex, hydration, or fatigue. Further investigation of the influence of diet and acute carbohydrate consumption is warranted. However, studies should first directly examine the impact of the aforementioned confounding factors so that they can be effectively taken into account during the design and interpretation of research on this topic. Findings from such studies will work to reduce the likelihood that confounding effects arising from changing brain metabolism or autonomic modulation complicate the interpretation of TMS data.

## Figures and Tables

**Table 1:** Test statistics, effect sizes and *p*-values for all significant differences.

Measure	Timepoint	Test Statistic	Effect Size	*p*-Value
GLU_glu_	T1–T0	7.781	1.834	<0.001
	T2–T0	9.370	2.209	<0.001
	T3–T0	6.963	1.641	<0.001
GLU_water_	T1–T0	−3.044	−0.717	0.043
GLU_suc_	T1–T0	7.434	1.752	<0.001
MAP_all_	T1–T0	7.817	1.842	<0.001
	T2–T0	5.123	1.208	<0.001
	T3–T0	6.922	1.632	<0.001
AURC_all_ *	T1–T0	2.940	0.490	0.003
	T2–T0	2.983	0.497	0.003

AURC_all_: area under the recruitment curve averaged across all treatments, GLU: glucose data for each of the three drinks, MAP_all_: mean arterial pressure averaged across all treatments * indicates that effect test statistics and effect size are derived from Wilcoxon’s test (i.e., *z* and *r*, respectively).

**Table 2:** Results of two-way ANOVAs with factors TREATMENT and TIME, and the grand coefficient of variation (CV) of each TMS measurement.

Measure	Grand CV (%)	Factor	*df*	F	*p*-Value
AURC ^¶^	60.8	TREATMENT	2,32	1.555	0.226
		TIME	2,32	10.429	0.001
		TREATMENT × TIME	4,64	2.290	0.069
SICI ^#^	80.8	TREATMENT	2,30	0.162	0.851
		TIME	2,30	2.640	0.088
		TREATMENT × TIME	4,60	1.712	0.200
SAI	44.6	TREATMENT	2,30	0.059	0.943
		TIME	2,30	0.118	0.889
		TREATMENT × TIME	4,60	0.268	0.897
LAI *	60.8	TREATMENT	2,32	2.663	0.085
		TIME	2,32	2.813	0.075
		TREATMENT × TIME	4,64	0.591	0.679

AURC: area under the recruitment curve, SICI: short-interval intracortical inhibition, SAI: short-latency afferent inhibition, LAI: long-latency afferent inhibition. * indicates data was square root transformed, ^#^ indicates log transformation, ^¶^ indicates data was ranked.

**Table 3:** Results from correlations between area under the recruitment curve (AURC) and capillary glucose (GLU) or mean arterial pressure (MAP).

Measure/Timepoint A	Measure/Timepoint B	Correlation Coefficient	*p*-Value
AURC T1_glu_	GLU T1_glu_	−0.432 *	0.261
AURC T1_glu_	GLU T2_glu_	−0.346	0.502
AURC T1_all_	MAP T1_all_	−0.127	0.978
AURC T1_all_	MAP T2_all_	0.078	0.996
AURC T2_glu_	GLU T2_glu_	−0.366	0.440
AURC T2_glu_	GLU T3_glu_	−0.492 *	0.144
AURC T2_all_	MAP T2_all_	0.017	1.000
AURC T2_all_	MAP T3_all_	−0.167	0.941
Ave AURC T1_glu_ & T2_glu_	Ave GLU T1_glu_, T2_glu_ & T3_glu_	−0.351	0.283
Ave AURC T1_all_ & T2_all_	Ave MAP T1_all_, T2_all_ & T3_all_	−0.089	0.924

Correlations were tested between relative change in AURC and GLU for the adjacent timepoint on the glucose visit only (T1_glu_, T2_glu_ and T3_glu_). Mean arterial pressure correlations were calculated for treatment-averaged data at each timepoint (T1_all_, T2_all_ and T3_all_). Correlations between the average change across all post-drink measures were also examined (bottom). All correlations were carried out using Pearson’s r unless data were not normally distributed, in which case Spearman’s rho (indicated by *) was used. Bonferroni correction was applied to all correlations.

**Table 4:** Results from preliminary ANOVAs confirming the presence of inhibition and no differences between T0 data for each measure.

Measure.	Factor	*df*	F	*p*-Value
AURC	TREATMENT (T0)	2,34	0.162	0.851
SICI	TREATMENT (T0)	2,32	0.206	0.732
Water	PATTERN	1,16	14.902	0.001
	TIME	2,32	0.463	0.560
	PATTERN × TIME	2,32	1.348	0.269
Sucralose	PATTERN	1,15	40.673	<0.001
	TIME	2,30	1.381	0.267
	PATTERN × TIME	2,30	3.372	0.069
Glucose	PATTERN	1,17	28.103	<0.001
	TIME	2,34	4.191	0.024
	PATTERN × TIME	2,34	1.384	0.264
SAI	TREATMENT (T0)	2,34	0.325	0.725
Water	PATTERN	1,16	49.518	<0.001
	TIME	2,32	0.002	0.998
	PATTERN × TIME	2,32	0.664	0.552
Sucralose	PATTERN	1,17	52.058	<0.001
	TIME	2,34	0.892	0.419
	PATTERN × TIME	2,34	0.345	0.710
Glucose	PATTERN	1,17	24.709	<0.001
	TIME	2,34	0.477	0.625
	PATTERN × TIME	2,34	0.100	0.905
LAI	TREATMENT (T0)	2,34	0.458	0.637
Water	PATTERN	1,16	16.100	0.001
	TIME	2,32	0.214	0.809
	PATTERN × TIME	2,32	2.256	0.121
Sucralose	PATTERN	1,17	28.422	<0.001
	TIME	2,34	0.858	0.414
	PATTERN × TIME	2,34	0.017	0.983
Glucose	PATTERN	1,17	8.101	0.011
	TIME	2,34	2.978	0.064
	PATTERN × TIME	2,34	1.024	0.370

AURC: area under the recruitment curve, SICI: short-interval intracortical inhibition, SAI: short-latency afferent inhibition, LAI: long-latency afferent inhibition.

## Supplementary Materials

The following are available online at https://www.mdpi.com/2076-3425/9/12/339/s1, Table S1: AURC, Table S2: SICI MEP size and SICI ratio, Table S3: SAI MEP size and SAI ratio, Table S4: LAI MEP size and LAI ratio, Table S5: Glucose, Table S6: Mean Arterial Pressure.
